# Physiological Activities of the King of Bitters (Andrographis paniculata): A Review

**DOI:** 10.7759/cureus.43515

**Published:** 2023-08-15

**Authors:** Sweza Bhaisare, Swanand Pathak, Vaishnavi V Ajankar

**Affiliations:** 1 Clinical Research, Datta Meghe Institute of Higher Education and Research, Wardha, IND; 2 Pharmacology, Jawaharlal Nehru Medical College, Datta Meghe Institute of Higher Education and Research, Wardha, IND

**Keywords:** anti-inflammatory, antioxidant, antibacterial, kalmegh, andrographis paniculate

## Abstract

*Andrographis paniculata* is a versatile tropical plant that is considered to have many essential medicinal properties. Plants can combine an open scope of the natural active compound that carries out significant organic roles and are used as pharmaceuticals. Herbal plants have therapeutic and prophylactic properties, so scientists are concentrating on developing crude drugs to make people's lives easier. Medicinal plants are the primary sources of therapeutic remedies for various ailments. These potential medicinal effects are primarily attributed to their active phytoconstituents. The importance of plants has been well understood by society and is well documented by scientists who have attached medicinal values to plants since ancient times. *A. paniculata* has been used as a herbal remedy for treating the upper gastrointestinal tract and upper respiratory tract, fever, herpes, diabetes, and other chronic illness. The *A. paniculata* treats a wide range of diseases in traditional medicinal systems, and its intended benefits must be evaluated. Depending on different plant studies, this review aims to discuss the medicinal values, pharmacological activity, and toxicity profile of *A. paniculata* like antibacterial, antioxidative, antidiabetic, anti-inflammatory, and anti-cancer activity for successful plant usage. In order to address the forthcoming research opportunities in the future, there is a need to establish a means of bridging the existing gap. It underscores the compilation of extensive pharmacological uses in order to establish the potential of *A. paniculata* as a versatile medicinal agent.

## Introduction and background

Plants have been historically utilized to heal various ailments, and *Andrographis paniculata* is one of them, with antibacterial and antioxidant activities. *A. paniculata* belongs to the family Acanthaceae and is commonly known as the 'king of bitterness’ [1]. It is an annual and branching plant with a height of 60-70 cm^2^, and glabrous surrenders to a length of 8 cm long, 2.5 cm expansive, and white bloom with petals having purple spots on them. The stem is dark green, 2-6 mm in diameter, and the seeds are small in size, yellowish-brown in color, and have a bitter taste. The plant's capsule is erect and direct elliptical, measuring 1-2 cm long and 2-5 mm wide, compressed, longitudinally grooved on broad faces, sharp at both ends, and sparsely glandular-hairy. Subquadrate seeds are exceedingly tiny [2]. The plant is significant for its diterpenoids acquired from the developed plants, which ordinarily develop through the seeds.

It is grown broadly in India, Sri Lanka, Java, Pakistan, Brunei, Thailand, Indonesia, and Malaysia. In India, it is found in Chennai, Orissa, Bihar, Jharkhand, Andhra Pradesh, Assam, Kerala, Karnataka, Uttar Pradesh, and Tamil Nadu. The plants are more suitable for high-yield and commercial cultivation. It is one of the most regularly involved rejuvenating plants in the ayurvedic, Siddh, and Unani frameworks of drugs as a home solution for different sicknesses in the Indian customary framework as well as in ancestral medicinal applications. It tends to be tracked down in a wide assortment of environments, for instance, fields, slopes, badlands, ranches, dry or wet terrain, drifts, and even the side of the road [3].

Some ayurvedic preparations contain *A. paniculata* which can be utilized to cure a variety of ailments and illnesses such as pathyadi kwacha churna, Nimbadi kwacha churna, Argvadhadi kwacha churna, tikka ghrita, bhunimbadi kwacha, Bhunimabadya ghrita, and Bhunimba dyashtadashanga kwacha. The synthetic constituents present in *A. paniculata* are andrographolide, panicolindefinene glucoside, panicolin, neoandrographolide, andrographidilnes, chlorogenic, myristic acid, homoandrographolide, andrographolide, andropanoside, etc. [4].

The different measurement types of *A. paniculata* that can be used as therapeutic dosage forms are as follows: juice for about 5-10 mL, powder for about 1-3 g, decoction for up to 20-40 mL, and liquid extract for up to 0.5-1 mL. The *A. paniculata* plant possesses activities like antimalarial [5], antioxidant and anti-inflammatory [6], anti-hepatic [7], antihyperglycemic [8], antibacterial [9], antipyretic [10], antihypertensive [11], anti-HIV [12], and anticancer [13], which are expected to be used for cholera, typical cold, influenza, poisons from the body, acidity, piles, dysentery, liver problems, gonorrhea, bite, fertility effect, and protection of the liver and gallbladder. The taxonomical classification of Kalmegh is shown in Table 1 [14].

**Table 1:** The taxonomical classification of Kalmegh.

1.	Kingdome	Plantae
2.	Division	Angiospermae
3.	Family	Acanthaceae
4.	Subfamily	Acanthoideae
5.	Genus	Andrographis
6.	Species	A. paniculata

Cultivation

Local to India, the plant favors a blistering, sticky environment with exposure to sunlight. Kalmegh can be created on a broad assortment of soils, from mineralized soil to medium ripeness, and begins blooming with the balance in temperature after the end of the storm. For cultivation, the site should be carefully prepared by repeatedly plowing to pulverize the dirt, first at the establishing stage and then 40 days after the manor. The utilization of 5 kg Azospirillium and 5 kg Phosphobacteria per hectare has likewise given excellent results. The field should be clear of weeds because it is a herbaceous plant. It is a robust plant and has not been harmed by any nuisance or infection [3]. Using *A. paniculata* suspension cultures, maximum andrographolide production can be achieved in a short period of time.

## Review

Methodology

The methodology for this review article includes a comprehensive literature search of academic databases, such as PubMed, Web of Science, and Scopus, to identify relevant articles, reviews, and reports that were identified in various previous studies. The search encompassed articles published from 2000 to the present. It utilized specific keywords such as "Physiological Activities," and "*A. paniculata*." Relevant articles, reviews, and reports addressing the research question are selected based on the inclusion and exclusion criteria. The inclusion criteria required that the articles be published in the English language, report on the physiological activities of the king of bitters, *A. paniculata*, in both observational and interventional studies, be published from 2000 to the present, and not be duplicated. Exclusion criteria included duplicate articles, articles in languages other than English, articles published in non-peer-reviewed journals, and articles published before 2000. 

Antibacterial properties

In various nations, the death rate is elevated because of irresistible bacterial illnesses. Salmonella, Clostridium, *Staphylococcus aureus*, *Eschericia coli*, Pseudomonas, and other microbes are some of the different pathogens that cause the infrequent contaminations of bacterial infections [15]. Furthermore, this organism can cause various diseases like cholera, tuberculosis, pneumonia, flu, measles, typhoid, intestinal sickness, legionnaire's illness, anthrax, and diarrhea, which can influence the human body at different levels. This creature can survive and flourish in a variety of environments. Anti-infection medicines are used to treat bacterial illnesses, which can be difficult to treat, ludicrous, and may also have some unintended consequences for the patient who has a location in other countries. These agents are substrates, inducers, and inhibitors of drug metabolic enzymes and transporters. However, bacteria develop resistance to antibiotics after some time. Recent research using different anti-infection agents suggests that more significant doses or prolonged infusions of some medications may boost the likelihood that they will work against some resistant pathogens.

Consequently, specialists progressively direct their concentration toward natural items for new antimicrobial stains. Root, stem, and leaf extraction can be utilized to hinder the development of all bacteria. The crucial constituent present in Kalmegh is andropholide -- Andrographolide, a lactone diterpene (Kumar et al. [16]). Concentrated water and methanol focus on *A. paniculata* leaves, stem, root, and entire plant to fight bacteria (*Staphylococcus aureus*, *Bacillus subtilis*, *Escherichia coli*, *Klebsiella pneumonia*, and *Proteus vulgaris*) using an agar well dissemination strategy. In Table 2, it is clear that many studies show the antibacterial activities of *A. paniculata*. In most studies, the leaves were found to be the most exciting part of isolating an antibacterial compound.

**Table 2:** Several demonstrations of antimicrobial investigations. GC-MS, gas chromotography-mass spectrometry

Organism affected	Plants part used	Extraction method	Extracts obtained
*Mycobacterium smegmatis*, *Staphylococcus aureus*, *Bacillus subtills*, *Pseudomonas aeruginosa*, *Eschericia coli* [17]	Leaf	Disc diffusion method	-
*Bacillus pumilus*, *B. subtilis*, *E. coli*, *Proteus vulgaris* [18]	Root	Agar cup plate method	Hexane, methanol
*P. vulgaris*, *S. aureus*, *P. aeruginosa*, *E. coli*, *B. subtilis* [19]	Leaf	Agar well diffusion method	Ethanol, methanol
*Staphylococcus epidermidis*, *B. subtilis*, *S. aureus*, *Enterobacter faecalis*, *Enterobacter cloacae*, Klebsiella pneumoniae, *Salmonella typhimurium*, *E. coli,* *P. aeruginosa* [20]	Aerial part	Agar well diffusion, GC-MS	Chloroform

Antioxidant properties

An oxidation response hypothesis states that oxygen responds inside the body and delivers results called free radicals. A free radical is an oxygen particle that has lost an electron. Because O2 is the final electron acceptor in the ATP-producing electron flow system. Cell reinforcements are materials that have extremely loose bonds in response to shattering properties. Practically all organic entities are safeguarded against less excessive damage by the existence of impetuses or blends, for example, ascorbic acid, tocopherols, and glutathione. Oxidation can cause diseases such as diabetes, bromhidrosis, hyperhidrosis, thyroid conditions, liver dysfunction, kidney failure, heart stroke, cancer, and Alzheimer’s heart disease [21]. A few engineered cell reinforcements, for example, butylated hydroxyanisole (BHA) and butylated hydroxytoluene (BHT), have limited use in food sources as they are believed to be cancer-causing and forestall lipid oxidation in fish feeds and essential unrefined substances. 

Verma and Vinayak [22] investigated the effect of *A. paniculata* aqueous extract on the antioxidant defense system in lymphoma-bearing AKR mice. It appears that the aqueous extract of *A. paniculata* was more effective than Doxorubicin in terms of its effect on catalase, superoxide dismutase, glutathione S transferase, and lactate dehydrogenase activities in mouse liver. Tripathi and Kamat [23] investigated aqueous extracts for antioxidant activity using rat liver subcellular organelles as a model system. They discovered that the section is a potent antiradical agent with multiple pathophysiology oxidant effects.

Antidiabetic activity

Andrographolide is an active bioactive synthetic constituent in *A. paniculata* that has antidiabetic potential. Andrographolide is the major labdane diterpenoid. The antihyperglycemic action was studied in STZ-diabetic rats, and the result proposed that andrographolide can increase glucose utilization to lower plasma glucose in diabetic rats lacking insulin [24]. Zhang and Tan [25] examined the antihyperglycemic property and figured out that the ethanolic concentrate may likewise lessen oxidative pressure in diabetic rodents. Yu concluded that *A. paniculata* was responsible for antihyperglycemic action in 2003.

Anti-inflammatory activity

Inflammation is the immune system's normal reaction to injury and sickness. Provocative synthetic substances in the circulation system work to safeguard your body from unfamiliar trespassers like microscopic organisms and infections. At the point when somebody is harmed, a confined inflammatory reaction assumes a fundamental role in the healing system. It is explained that dehyroandrographolide, followed by neo andrographolide, and andrographolide, essentially decreased irritation caused by receptors dimethyl benzene and adrenaline with normal reaction to injury and sickness. 

Liu et al. [26] studied the anti-inflammatory properties of neo andrographolide isolated from the medicinal herb *A. paniculata* in vivo and in vitro. Their results show that neo-andrographolide has an anti-inflammatory effect. Sheeja et al. [6] investigated the anti-inflammatory properties of the plant Kalmegh in vitro and in vivo; they found that the administration of a methanolic extract from *A. paniculata* prevented carrageenan-administered inflammation as compared to a control animal. In a double-blind clinical trial, Poolsup et al. [27] discovered that Kalmegh, either by itself or in combination, was more successful than placebo treatment and could be a substitute treatment for specific intense upper respiratory tract disease. All of the findings demonstrate that *A. paniculata* and its constituents have anti-inflammatory properties.

Anti-cancer activities

Cancer is a category of more than 100 diseases defined by unbonded cellular proliferation. Invasion of local tissue and distant metastases are also possible outcomes. It is the second-most significant reason for death around the world, after cardiovascular problems. Every year, cancer kills more than 6 million people around the world. In 2001, the United States recorded approximately 12,68,000 new cancer cases and 553,400 fatalities. Plants have been utilized as a source of medication for the treatment of infirmities since the Middle Ages. In the last part of the 90s, the WHO expressed that a significant portion of the total populace relies upon herbal-based treatments to balance the requirements of essential medical services.

During the process of in vivo testing, the research conducted by Rajagopal et al. demonstrates the intriguing pharmacological properties of andrographolide, a chemical compound, in the B16F0 melanoma syngenic and HT-29 xenograft models. The results indicate that andrographolide has noteworthy anticancer and immunomodulatory activities [28]. In B16F0 melanoma syngenic and HT-29 xenograft models, it is suggested that the isolated chemical andrographolide is a fascinating pharmacophore with anticancer and immunomodulatory effects. Researchers looked for naturally occurring chemicals that would cause leukemia cells to differentiate in a mouse study. Kalmegh was picked because it contained terpenes, similar to terpenes tracked down in different plants and known to trigger cancer cell differentiation. The findings showed that *A. paniculata* could induce cell differentiation in leukemia cells [29].

Anti-fertility and contraceptive effects

Anti-fertility medicines, also known as oral contraceptives, are drugs that limit fertility. These medications have an impact on and influence females' menstrual cycles and ovulation. Birth control tablets contain a combination of estrogen and progesterone. When an antifertility agent hinders fertilization, ovulation, implantation, kills the zygote, or includes termination of the fetus in females, it is considered active. In the male, it impairs spermatogenesis, suppresses testosterone or has an effect on organ gonadotrophin or sperm mortality. In many developing countries, population growth is now regulated. To inhibit gametogenesis, male rats were given dry ap leaf powder every day for around 60 days. The treatment brought about the gathering of glycogen and cholesterol in the testis and expanded the activities of lactate dehydrogenase in the testis and antacid phosphatase in the testis and ventral prostate. The outcomes propose a spermatogenic and additionally antiandrogenic impact on the plant [30]. Andrographolide sodium succinate was found to be viable for restraining human progesterone in investigations conducted on cultured human placental tissue. Even at the most outstanding levels examined, there was no deterrent effect on other typical human issues, and the study showed that the substance and plant are naturally contraceptive. It was found that there were no pregnant female mice who consumed *A. paniculata*-mixed food daily after mating with the untreated male of potential fertility, implying that AP has a contraceptive effect on female mice [31].

Toxicity

Animal investigations have repeatedly shown that *A. paniculata* is a low-toxic species. On the other hand, oral portions of Kalmegh might produce nausea, vomiting, and anorexia. This reaction seems to be caused by andrographolide’s harsh taste. The further active study, including lengthier clinical trials and the separation of its phytoconstituents, is needed to make Kalmegh a viable alternative resource in medical care. A portion of 1,500-2,000 mg of andrographolides was given for about 42 days to HIV-positive participants in the research. Despite some gains in CD4+, the study was cut short due to side effects [32].

## Conclusions

The immense writing study and exploratory outcomes investigation concludes that Kalmegh is a customary solution for different diseases and has been used as an antibacterial, antioxidant, antidiabetic, antifertility, and for liver problems and fever. For various conditions, some outcomes enriched with extracts and outlying substances have been seen in the public and worldwide business sectors. The isolated chemicals derived from Kalmegh extract have exhibited significant efficacy as agents for cancer prevention, relaxing effects, anti-inflammatory activity, parasite activity, antioxidant activity, antidiabetic effects, and anti-hypoglycemic effects.. According to an assessment of the literature, Kalmegh has a wide variety of pharmacological activities, whether in concentrate, powder, or separated compounds, with minimal antagonistic impacts. Albeit this survey is exceptionally encouraging for the utilization of the plant as a multi-reason restorative specialist, a few limitations presently exist in the current written works. This article discusses Kalmegh's pharmacological activity in both experimental and clinical settings.
